# Supplementary material for: Are carbon emissions trading and green financial instruments synergistic? -Comprehensive quantitative research based on content analysis
Source: PLoS One. 2024 Mar 7;19(3):e0298601. doi: 10.1371/journal.pone.0298601 (PMC10919601; doi:10.1371/journal.pone.0298601)
Supplement: S1 Data — (ZIP) [file pone.0298601.s001.zip › Data/地级市Z_F工作报告.zip!/╝¬┴╓/░╫╔╜╩╨/└┤╫╘╣1⁄2╓┌║┼í╛┬φ┐╦╩2╛▌═°í┐_2007.docx]

各位代表：

　　我受市政府委托，向大会报告工作，请予审议。并请各位政协委员提出意见和建议。

　　一、关于2006年工作的基本回顾

　　2006年，我们在省委、省政府和市委的正确领导下，认真贯彻落实科学发展观，狠抓项目建设、招商引资、企业改革、提速增效等重点工作，全面发展各项社会事业，努力解决人民群众切身利益问题，全市经济社会步入又好又快的发展时期，圆满完成了市五届人大二次会议确定的各项任务，实现了“十一五”的良好开局。

　　发展速度明显加快。全市完成地区生产总值191.9亿元，同比增长18.1%，比2002年翻了近一番；人均GDP达到1.45万元，居全省第四位。完成地方级财政收入7.8亿元，一般预算全口径财政收入15.7亿元，分别增长44.8%和22.8%，均比2003年翻了一番多。完成规模以上工业增加值65.7亿元，增长24.2%；实现净盈利5.9亿元，增长32.3%。实现农业增加值27.8亿元，增长6.7%。以144个试点村为重点，扎实推进新农村建设，投入帮扶资金4885万元。实现社会消费品零售总额66.1亿元，增长16.2%。

　　发展后劲明显增强。“项目落实年”活动成效显著，完成全社会固定资产投资130.7亿元，增长74%，比2003年翻了近两番。完成工业技术改造投资40.2亿元，增长103%，比2004年翻了两番。长青铁矿40万吨铁精粉、东圣焦化90万吨煤炭深加工等23个项目竣工或部分投产，白山热电公司2×300兆瓦机组、松江河梯级电站等104个续建项目进展顺利，金刚水泥熟料生产线、丽美坚实木复合地板等68个新建项目陆续开工建设，形成了加快总量扩张的强劲发展势头。全年创中国名牌产品2个、吉林省名牌产品3个、中国驰名商标2个、吉林省著名商标28个、使用地理标志保护产品专用标志先进企业3个，位于全省前列。

　　发展环境明显优化。经过不懈努力，我市被国务院批准为综合性资源型城市经济转型试点，可持续发展实验区通过国家17部委验收，江源县恢复市辖区体制，为更好更快发展创造了有利条件。公路建设完成投资11.8亿元，市区南环线、临江至八道沟、沿江至万良、长白山南坡旅游公路等一批重点项目相继竣工，全市乡镇实现了通油路。宇辉铁路正式投入运营。市区公用设施建设完成投资2.8亿元，是“十五”期间的1.8倍。通江大桥、月牙河、城市给排水管网改造和垃圾处理场、市区部分主次干道及巷道建设等一批重点工程顺利完工，城市功能不断提升，人居环境得到改善。通过市区共同努力，实现了创建省级卫生城市目标。

　　改革开放力度明显加大。全市完成企业改革62户，支付改制成本3亿多元，1万多名在册职工得到妥善安置。对外开放度和经济外向度进一步提高。积极参加省政府组织的各项经贸活动，自行组织了赴大连、丹东、香港、新加坡、韩国等地的招商活动，成功举办了第三届中国·白山国际矿泉节。全市实际引进域外资金38亿元，其中省外资金31.7亿元，分别增长27%和140%。引进了康师傅、江苏雨润、海南航空、吴太集团等一批战略投资者。实现外贸出口1.22亿美元，增长26.8%，首次突破1亿美元大关。

　　各项社会事业明显进步。与高等院校、科研院所签订了14个科技合作项目。申请国家、省科技发展计划项目30项。素质教育得到加强，学校布局更趋合理，办学条件进一步改善。全国“双拥”模范城创建工作扎实推进，文化拥军受到总政和民政部表彰。长白山满族文化博物馆实现主体封闭。建成了东北三省第一个“电话收听广播工程”。市中心医院住院大楼、全民健身活动中心投入使用。在省十五届运动会上，金牌总数和总成绩均列第三名。全面兑现了农村计划生育奖励扶助政策，推行了城镇独生子女父母退休后奖励政策。人口自然增长率控制在2.74‰。

　　人民生活明显改善。认真解决就业、社会保障、采煤沉陷区和棚户区改造等事关群众切身利益的实际问题。全年新增城镇就业岗位4.6万个，下岗失业人员再就业2.8万人，劳务输出2.1万人。共发放养老金、失业保险金、最低生活保障金8.22亿元，离退休职工、下岗失业人员和城镇困难群众的基本生活得到切实保障。棚户区、采煤沉陷区改造共拆迁68万平方米，开工187万平方米，回迁101万平方米，10540户居民喜迁新居。预计城镇居民人均可支配收入、农民人均纯收入分别达到9600元和3665元，增长10.5%和9.5%。

　　政府自身建设明显加强。自觉接受人大的法律监督、工作监督和政协的民主监督，及时办复人大代表建议和政协委员提案146件。“五五”普法教育全面启动。服务型、法治型、责任型、效能型政府建设逐步推进，政务、村务、厂务公开进一步深化。开展了“治庸、治懒、治散”专项行动，行政效率和服务质量得到提高。应急管理体系逐步完善，快速反应、协调联动的能力明显增强。深入开展了煤矿、非煤矿山、危险化学品、交通、消防等安全隐患整治，加强了对食品、药品的安全监管。加大了资源和生态环境保护力度。实现了连续27年无重大森林火灾。组织开展了一系列严打整治专项行动，治安状况进一步好转。畅通政府与人民群众的联系渠道，群众反映的一些热点、难点问题得到解决。

　　各位代表，这些成绩的取得，是全市人民在市委领导下，团结奋斗的结果。在此，我代表市政府，向全市广大工人、农民、知识分子、老同志和各级干部，向驻白山的解放军指战员、武警部队官兵和全市公安司法干警，向中省直企事业单位致以崇高的敬意！对市人大、市政协、各民主党派、工商联、人民团体和社会各界给予政府工作的大力支持和热情帮助，表示衷心的感谢！

　　回顾一年来的工作，我们也清醒地认识到：我市高科技含量、高附加值项目少，对资源依赖度较大，产业链条短，新兴产业发展缓慢。市场主体自主创新能力较弱，经营管理水平亟待提高。农业产业化缺少龙头企业带动，农特产品深加工能力较低。城市基础设施欠账仍然很多，部分区域供水、供电、供热和硬覆盖、内涝等问题急需解决。就业和再就业压力较大，社会保障和救助体系不够完善，困难群体比重偏高。安全生产事故频发，形势依然严峻。社会治安防控体系还需进一步加强。各级公务员队伍中还存在服务意识不强、工作作风不实、办事效率不高等突出问题，发展环境需要进一步改善。对这些问题，我们一定高度重视，认真加以解决。

　　二、关于全面贯彻市五次党代会精神

　　市五次党代会是在白山进入新的发展时期召开的一次承前启后、谋划未来的大会。全面贯彻落实会议精神，就是要以科学发展观为统领，以富民强市为主题，以改革开放为动力，全面推进资源型城市经济转型，提升传统产业，壮大新兴产业，做强加工制造业，加快结构调整和增长方式转变，经过五年努力，把白山建成工业强市、旅游大市、特产名城和宜居佳地，实现更好更快建设全面小康的长白山第一市的阶段性目标。

　　围绕工业强市建设，着力改造提升传统产业，培育壮大新兴产业，加快工业化进程，逐步形成工业比重大、增长速度快、竞争实力强的产业格局。重点建设“四个基地”：一是能源基地。到2011年，全市煤炭产量稳定在1000万吨，火电总装机达到125万千瓦，水电总装机达到130万千瓦以上，力争开工建设核电，形成煤炭、火电、水电、核电并举的能源产业体系，同时大力发展煤化工项目，实现原煤就地转化增值。二是矿产新材料基地。把矿产冶金业由原材料输出型向加工制造型转变，不断开发新产品、新工艺，发展粉体材料、稀土功能材料、轿车用新材料、电子信息材料、稀有金属材料等，延伸产业链条。到2011年，矿产新材料产业实现增加值24亿元，占全市规模以上工业增加值的10%。三是林浆纸和复合地板生产基地。坚持资源保护与开发并重，继续开发生产复合地板、高密度纤维板、纸浆等产品，并向高档家具、新型装饰材料等产品延伸。到2011年，纸浆、复合地板和人造板生产能力分别达到30万吨、2000万平方米和60万立方米。四是循环经济基地。充分利用我市的煤矸石、粉煤灰、铁矿尾矿等工业废弃物，发展水泥、氧化铝、纸浆添加剂、建筑砌块等项目，形成资源——产品——废弃物——再利用的循环经济链条。到2011年，循环经济实现增加值32亿元，占全市规模以上工业增加值的13.5%，把白山经济开发区建成国家循环经济示范园区。在加快四个基地建设的同时，努力将绿色食品、医药等产业培育成为新的支柱或主导产业。到2011年，全市规模以上工业增加值达到236亿元，比“十五”期末增长3.7倍，占GDP的比重达到49%。

　　围绕旅游大市建设，以创建全国优秀旅游城市为目标，进一步整合旅游资源，加强旅游基础设施建设，开发精品旅游线路，建设三大旅游板块，打造长白山旅游产业集群。一是以长白山西坡登天池、锦江大峡谷、温泉旅游度假区、露水河狩猎场、仁义风景区、杨靖宇殉国地为重点，建设松花江上游景观带；二是以长白山南坡登天池、鸭绿江大峡谷、望天鹅风景区、对朝跨国旅游为重点，建设鸭绿江景观带；三是以龙山湖、长白山迷宫、干饭盆、枫叶岭、溪谷、长白山满族文化博物馆为重点，建设环城景观带。到2011年，实现旅游业社会总收入48亿元，占GDP的比重达到10%，把白山建成长白山区域旅游集散中心和著名旅游胜地。

　　围绕特产名城建设，充分发挥比较优势，突出可再生资源的开发利用，通过产业接续、产业集聚、三次产业链接，实现特色产业的快速发展。一是大力发展矿泉水产业。进一步提升娃哈哈、农夫山泉、泉阳泉等名牌矿泉水产能，支持康师傅、吴太集团等矿泉饮品企业加快发展。到2011年，矿泉水生产能力达到500万吨，打造具有世界竞争力的国际矿泉城。二是加快发展山珍绿色食品产业。顺应天然、营养、无公害的消费趋势，深度开发人参、西洋参、林蛙、山珍野果、山野菜等特色产品，建设一批规模较大的山珍绿色食品加工企业，构建山珍绿色食品产业集群。三是推进医药产业产品升级。坚持中药、西药和生物制药并举，打造以天然药、中成药和高档保健品为主体，集保护、种植、研发、加工于一体的医药产业体系。重点建设刺五加、北五味子等八大名贵中药材保护基地和人参、西洋参等八大中药材GAP种植基地，扶持拥有2项国家专利的天风药业和百年老字号的抚松制药等15户骨干企业，开发一批具有自主知识产权的新药品种。到2011年，全市特色产业实现增加值105亿元，占GDP的比重达到21.8%，把白山建成长白山特产加工和输出名城。

　　围绕宜居佳地建设，重点实施“四项工程”：一是立体交通网络建设工程。未来五年，全市要完成交通建设投资107亿元，建成白山至长春、沈阳(丹东)的高速公路以及长白山机场、靖宇至松江河铁路、环长白山旅游公路等交通基础设施，形成立体、快捷的交通网络。二是棚户区改造工程。实行新区开发与旧城改造并举，在高起点开发江北新区、改造南山区的同时，完成市区、县城棚户区和煤矿棚户区改造任务，切实改善人居环境。三是“畅绿净亮美”工程。以创建国家卫生城市为目标，加大基础设施建设和环境整治力度，彻底改变城市面貌。建设包括江源区在内的市区外环路。城区逐步实现全部巷道硬覆盖，增加绿覆盖面积，完善供电、供水、供热、供气、污水及垃圾处理等公用设施。加快美化、亮化建设，提高城市品位和形象。四是市区江河改造工程。结合浑江干流堤防建设，修建八道江、江源城区沿江带状公园，并拦江蓄水，形成新的景观带。以治脏、排涝为重点，对两区内河彻底整治，结合旧城改造和新区建设，引水入小区，逐步形成溪在小区流、鱼在水中游、人与自然和谐的山水园林城市。

　　为完成市五次党代会提出的目标和任务，市政府工作要更加注重科学发展，更加注重改革创新，更加注重艰苦创业，更加注重协调发展，更加注重和谐文明。科学发展，就是要坚持依托资源而不依赖资源的发展理念，快速扩充总量，调整优化结构，转变增长方式，提高运行质量，实现更好更快地发展。改革创新，就是要不断推动体制和机制创新，努力提高自主创新能力，增强发展的动力和活力。艰苦创业，就是要营造更加良好的创业环境，激发全市人民的创业热情，让一切创造财富的源泉充分涌流，使人民生活更加殷实富裕。协调发展，就是要实现经济与社会良性互动，城市与乡村一体发展，市、县(市)区共同进步。和谐文明，就是要坚持以人为本，统筹协调各方面利益关系，努力建设和谐白山、文明白山、法治白山、平安白山、绿色白山，让改革与发展的成果惠及全市人民。

　　三、关于2007年重点工作安排

　　今年是我市深入实施“十一五”规划、全面贯彻落实市五次党代会精神、加快推进经济转型的重要一年。市政府工作的总体要求是：以科学发展观为统领，以更好更快发展为主题，以推进经济转型为主线，以投资拉动为突破口，突出工业立市、工业强市，着力调整经济结构和转变增长方式，着力促进资源节约和加强环境保护，着力推进改革开放和自主创新，着力扩充经济总量和壮大经济实力，着力促进社会发展和解决民生问题，不断开创振兴白山、富民强市的新局面。今年经济社会发展的主要预期目标是：全市地区生产总值增长20%以上，其中一、二、三产业增加值分别增长6.7%、24.7%和13.2%，全口径和地方级财政收入与经济保持同步增长，全社会固定资产投资增长53%，社会消费品零售总额增长10%，外贸进出口总额增长20%，城镇居民人均可支配收入、农民人均纯收入分别增长12%和10%，人口自然增长率控制在4.7‰以下，城镇登记失业率不超过4.5%。在实际工作中，我们要力争超额完成任务，重点抓好以下八个方面的工作。

　　(一)深入实施项目带动战略，快速扩充经济总量。认真落实国家宏观调控政策，继续加大投资拉动力度，优化投资结构，以大投资催生大项目、集聚大产业，提高项目建设质量和投资效益。全年完成固定资产投资200亿元，其中工业投资完成100亿元以上。白山热电、金刚水泥、丽美坚复合地板、康师傅系列饮料、恒巨铝车轮等44个项目建成投产。松江河梯级电站、大横路钴矿、天风药业、板石矿业等42个续建项目加快进度。林浆纸一体化、板庙子黄金、雨润集团肉食加工等62个项目开工建设。抓好靖宇核电站、2000吨单晶硅、松江河抽水蓄能电站等重大项目前期工作。打破行政区划界限，引导项目向工业集中区和矿泉水、人参、硅藻土、复合地板等特色工业园区集聚，形成产业集群。开展“地质勘探年”活动，逐步探明矿藏储量。强化政府对优势资源的掌控力度，组成矿产新材料、林产品深加工、绿色食品、医药等8个专业项目谋划组，对有意向的大项目紧盯不舍，对有合作希望的大项目紧追不舍，对有潜力的大项目紧挖不舍，谋划生成一批成长性好、附加值高、带动作用强的工业项目，形成滚动实施、梯次推进的项目建设新局面。全力支持中省属企业上项目。加快发展民族地区经济。

　　(二)切实转变增长方式，提高经济运行质量。靠改革激发活力、技改提升能力、创新提高竞争力、名牌扩大影响力、要素平台增强支撑力，促进工业经济提速增效。推动改制企业加快建立现代企业制度，解决好特殊工种认定、退休职工医保及取暖费等遗留问题，全面启动厂办大集体企业改革，力争上半年完成任务。继续加大技术改造力度，完成投资60亿元，增长50%。推进经济要素向高成长性的企业倾斜，逐步将天风药业、白山热电、康师傅矿泉水等企业培育成销售收入超10亿元的企业集团。坚持引进先进技术与自主创新相结合，增加科技投入，全年引进科技成果12项以上，帮助5户企业建立研发中心。争创一批国家、省名牌产品及驰名、著名商标，新增一批地理标志保护产品和国家免检产品。加强企业家队伍建设，引导企业不断提高管理、融资、营销和研发能力。鼓励商业银行加大对地方经济发展的支持力度，提高市城市信用社股份有限公司的信贷能力，增强市中小企业担保公司担保能力，组建各县(市)中小企业担保公司。提高国有资产投资公司运营水平，使其成为产权流转和贷款担保的平台。选择几户重点企业进行上市辅导，争取尽快上市。进一步加强资源节约和生态保护，万元工业增加值综合能耗下降8%，主要工业污染物化学需氧量和二氧化硫排放量分别降低2%。规模以上工业增加值增长24%，实现净利润增长22%，新增规模以上工业企业45户。

　　(三)加快发展现代农业，扎实推进新农村建设。充分发挥丰富的林下资源优势，坚持壮大特色优势产业与发展县域经济、推进工业集中区建设相结合，培育壮大人参、林蛙、中药材、食用菌和特种养殖等产业。稳步压缩伐树种参面积，扩大林下种参和参后轮作面积，逐步发展人参期货，争取建设国家级人参检测中心，打响长白山人参品牌。加快五味子、桔梗等GAP基地认证，推进中药材生产基地标准化、规模化。以松野黑猪养殖为重点，加快发展精品畜牧业。扶持国姿北药、万良人参市场、大阳岔食用菌公司等龙头企业尽快做大做强。新增省级龙头企业6户、市级龙头企业15户，充分发挥其带动功能，全面提升农业产业化水平。加快发展农村经济合作组织，年内达到450个，带动农户2.5万户。引进北京三安生物农业安全食品生产技术，创新订单农业组织形式，积极探索依靠高新技术促进农民增收的发展模式。加强农业实用技术、致富技能培训，鼓励和支持农民外出打工，发展劳务经济，提高农民增收能力。高标准完成试点村镇建设总体规划，培育一批有产业支撑、有鲜明特色的先进典型，加快新农村建设步伐。继续加大对农村公共事业的投入，投资1000万元，完成28处农村人畜安全饮水工程；投资2亿元，完成30个村的公路建设，八道江区和靖宇县在全市率先实现村村通油路。精心组织好第二次农业普查。认真做好第七次村民委员会换届工作。全面深化农村综合改革，逐步化解乡村两级不良债务，加强村级财政转移支付资金和涉农服务性收费的监管，切实减轻农民负担。集中整治村容村貌，实施千里河道清洁工程，着力解决“脏、乱、差”问题。深入开展“除陋习、树新风”活动，切实提高农村精神文明建设水平。

　　(四)培育壮大旅游产业，带动服务业加快发展。设立旅游产业发展专项基金，编制完成全市旅游总体规划和重点景区控制性详细规划，打造以生态观光、冰雪娱乐、边境风情、红色旅游和科考探险为特色的旅游产品，加大宣传推介力度，办好各项节庆活动，充分展示白山旅游新形象。加强与周边地区特别是长白山管委会在旅游资源保护、基础设施建设和旅游市场开发等方面的合作。加快推进望天鹅、龙山湖等景区的综合开发。完成温泉资源整合及规划设计，开工建设长白山国际温泉旅游度假区。以设施完善、卫生达标、服务规范为目标，集中开展旅游环境综合整治，切实提高接待能力和服务质量。实现旅游业总收入11.5亿元，增长20%。坚持传统服务业和现代服务业一起抓，生产性服务业和消费性服务业一起上，着力发展现代物流业，启动运行市物流中心，培育具有白山特色的专业物流市场，逐步建成人参、绿色食品、中药材和林木产品区域物流平台，进一步完善长白山山货市场、万良人参市场功能，加快建设长白山国际商业批发中心、星泰农贸市场和江北建材商贸城。大力发展信息、金融、中介、保险、法律等新兴服务业。

　　(五)强力推进招商引资，全方位扩大对内对外开放。抓住国际产业转移和沿海发达地区资本扩张的有利契机，推动招商引资再上新台阶，不断提高经济外向度。积极参加“中俄国家年”、“东博会”等系列经贸活动。重点选择东北、华北、胶东半岛以及东北亚、东南亚等国家和地区，有针对性地开展多种形式的招商引资。组织好“香港经贸周”活动，办好国际矿泉节、长白山人参节和构建东北东部经济带论坛。进一步拓宽思路，实行开发区园区平台招商、资源打捆招商等有效方式，提高招商引资的吸引力和成功率。全面落实县(市)区和部门招商引资目标责任制，严格兑现奖惩。全市引进域外资金65亿元，增长71%，其中引进外资4000万美元，增长66%。适应外贸形势变化，鼓励企业到俄罗斯、朝鲜等国家合作开发林木、矿产等资源，建设境外原材料基地，扩大对外劳务输出规模。建设复合地板、人参、碳化硅、硅藻土等出口商品基地，增强企业出口能力。加快对朝互市贸易区建设，积极开展边境贸易。全市完成外贸出口总额1.46亿美元，增长20%。

　　(六)掀起全民创业高潮，推动民营经济腾飞。在全市深入实施民营经济腾飞推进工程，采取放开、扶持、带动、保护等有效措施，坚持改制发展一批、招商引进一批、创业催生一批、基地孵化一批、扶优促强做大一批，快速扩充民营经济总量。鼓励机关干部离职、挂职领办、创办企业，扶持广大群众发展个体经营，支持和帮助现有民营企业做大做强，在全社会形成百姓创家业、能人创实业、干部创事业、企业家创伟业的浓厚氛围。坚持非禁即入原则，在资源配置、立项审批、土地使用、信贷投放、对外贸易等方面，全力支持民营经济发展。从创业扶持、人才培养、技术支持、信息咨询等方面，完善民营经济社会化服务体系。积极争取省创业孵化基地建设专项资金，加快中小企业孵化基地建设。建立中小企业融资服务平台，完善信用担保体系。实施“百户民营企业成长工程”和“20强民营企业评选活动”，树立一批创业典型，使优秀民营企业经营者在政治上有地位、社会上有声誉、经济上有发展。严肃查处吃拿卡要、刁难设障、乱罚乱收等违规行为，保障民营企业的合法权益。全市民营经济总收入和上缴税金分别增长22%，规模以上企业增加到165户，销售收入超亿元企业增加到18户。

　　(七)加快基础设施建设，完善区域发展环境。推进长白山机场建设，确保奥运会前通航。公路建设总投资20亿元以上，完成环长白山旅游公路、马鹿沟至八号闸、老岭至临江等项目年度施工计划。争取营城子至抚松高速公路和松江河至长白公路等重点项目开工建设。做好靖宇至松江河铁路前期工作，争取年内开工。完成横山水利枢纽工程，双河电站投产发电，开工建设抚松枫林、老松江电站。完成白山经济开发区、江源工业集中区、长白山机场供电工程和长白220千伏输变电工程。

　　统筹八道江区、江源区规划，完成城市建设投资22亿元。开工建设连接市区南北线的2座跨江大桥和向江路大桥，建成通江路、靖宇路公铁立交桥，维修改造彩虹桥。完成南平街、苇塘路等25条街路建设，打通市中心区所有半截路，初步形成快捷通畅的城市交通网络。全部完成采煤沉陷区综合治理和已开工的城市棚户区改造工程，新开工75万平方米市区棚户区和100万平方米煤矿棚户区改造工程，加快建设市区集中供热三期和管道燃气工程，全面启动各县(市)城区棚户区改造。新建和改造市区路灯870组，主要街路安装楼形灯、射灯和霓虹灯，加快浑江干流市区段灯饰带建设，形成立体夜色景观。开工建设满族风情园和翠柏公园，完成儿童公园综合改造。抓好北山植物园、市区街路和居民小区绿化美化，新建绿地20万平方米、植树100万株。切实强化城市管理，整合城市综合执法资源，加大城市管理和市容环境整治力度，为创建国家级卫生城夯实基础。

　　(八)坚持以民为本，努力建设和谐白山。继续把扩大就业摆在更加突出的位置，认真落实就业再就业扶持政策。加强创业和职业技能培训，提高就业再就业能力。采取政府提供公益性岗位等措施，及时解决“零就业”家庭等困难群体就业问题。跟踪项目建设抓好岗位开发和对接。积极扩大劳务输出。全年新开发就业岗位3.6万个，城镇新增就业2.9万人。强化劳动者权益保障，严格执行最低工资标准，严肃查处拒不投保、欠发工资等违法行为。以非公有制企业扩面征缴为重点，提高养老、失业保险基金支撑能力。着力解决被征地农民社会保险问题。城镇职工基本医疗保险扩大到43万人，城镇居民住院医疗保险扩大到22万人。全面启动新型农村合作医疗。进一步扩大工伤和生育保险覆盖面。完善城乡社会救助体系，强化“应保尽保”动态管理，落实省定城乡居民低保补差标准，逐步实现城乡最低生活保障全覆盖。进一步健全困难群体包保责任制，重点解决他们在基本生活、就医、住房、看病、子女上学等方面的实际困难。加快发展社会福利和慈善事业，完成全市农村社会福利服务中心建设。

　　继续加大对各项社会事业的投入。深化教育改革，全面推行素质教育。筹建长白山职业技术学院，异地新建市实验小学。全部免除农村义务教育阶段学生学杂费。积极发展公益性文化事业和经营性文化产业，长白山满族文化博物馆年内开馆。加大文化新闻出版市场整治力度，严厉打击网络侵权盗版和网吧非法接待未成年人行为。加强公共卫生体系建设，完善农村三级卫生服务网络和以社区服务为基础的新型城市卫生服务体系，有效解决群众看病难、看病贵问题。继续抓好广播电视“村村通”工程，加快老岭微波站改造，完成广播电视中心大楼建设。全力备战全国第十一届冬季运动会，积极筹备明年全市第六届运动会，大力开展群众性体育活动。继续稳定低生育水平，提高出生人口素质。深入开展“双拥模范城”创建工作。依法加强对宗教事务的管理。认真落实《白山市优待老年人规定》。切实维护妇女儿童的合法权益。

　　健全党政军警民合力治边机制，确保边境安全稳定。切实防范和打击邪教组织、敌对势力的捣乱破坏和颠覆渗透活动，维护政治安定。加强社会治安综合治理，严厉打击各种违法犯罪活动，进一步增强人民群众安全感。严格落实安全生产责任制和责任追究制，切实加强煤矿、非煤矿山、交通运输、危险化学品、建筑施工、消防、校园和食品药品卫生等安全管理，建立重大危险源和重大事故隐患动态管理系统，增强公共安全保障能力，坚决遏制重特大事故的发生。加强应急管理，完善预案体系，提高应对突发公共事件的能力。

　　四、关于加强政府自身建设

　　按照胡锦涛总书记关于倡导“八个方面良好风气”的要求，努力加强政府自身建设，全面提高公务员队伍素质，建设一个心齐气顺、风正劲足的政府机关。

　　转变政府职能，创新管理方式。用创新的思维谋划工作，创新的方法落实任务，创新的手段提高效能。结合公务员登记和事业单位人事制度改革，进一步优化政府机构设置，理顺部门之间的职能分工，鼓励机关内部机构调整和人员优化组合。全面推行行政审批权相对集中改革，简化审批程序和环节，进一步强化市、县两级政务大厅服务功能，把建设工程、政府采购、产权交易等招投标活动纳入各级政务大厅规范运行。扩大部门预算、国库集中支付改革试点和政府采购范围，全面完成非税收入管理体制改革。

　　改进工作作风，提高行政效能。牢固树立抓效能建设就是抓核心竞争力、就是抓发展、就是抓政府行政能力的机关建设理念。通过各种行之有效的教育方式和培训手段，使每位机关干部都能带着责任去工作，带着使命去工作，带着激情去工作，今天能解决的问题决不拖到明天，自己能解决的问题决不推给别人。从市政府做起，从市直机关做起，推进工作重心下移，重实干、办实事、求实效，围绕项目建设、招商引资等重点工作，围绕城市拆迁、土地征用等人民群众最关心、最直接、最现实的利益问题，认真解决各类矛盾和困难。继续完善、细化绩效考核指标体系，改进考评方法，坚持严管厚爱，奖优治庸罚劣。

　　坚持民主决策，推进依法行政。建立健全公众参与、专家咨询论证和行政机关决定相结合的决策机制，进一步完善决策程序。重大决策必须以基础性、战略性研究或发展规划为依据，涉及长远发展的重大事项必须认真组织专家论证，涉及人民群众切身利益的问题必须广泛征求群众意见。进一步完善政府工作制度，认真落实行政执法监督和错案责任追究等有关规定，做到文明、规范、公正执法。各级政府和部门要自觉接受人大的法律和工作监督、政协的民主监督以及社会公众监督和媒体舆论监督，同时加强政府内部的监察、审计等专项监督，全面促进依法行政。

　　坚决反腐倡廉，防止权力滥用。坚持标本兼治、综合治理、惩防并举、注重预防的方针，建立健全教育、制度、监督并重的惩治和预防腐败体系，做到用制度管权、按制度办事、靠制度管人。加大对工程建设、土地出让、产权交易、医药购销、政府采购等领域商业贿赂的查处力度。继续深入开展民主评议政行风活动，坚决纠正损害群众利益的不正之风。认真落实整治发展软环境的各项规定，严肃查处“涉软”案件。努力营造风清气正的行政环境，建设人民群众满意的政府机关。

　　各位代表！白山正处在增强发展基础、积聚发展能力、加快经济转型、实现崛起振兴的关键时期。让我们在省委、省政府和市委的正确领导下，大力弘扬“向前向上、求富求强”的白山精神，以崭新的面貌、昂扬的斗志，向着建设全面小康的长白山第一市宏伟目标奋力迈进！
